# In-Vivo Assessment of Femoral Bone Strength Using Finite Element Analysis (FEA) Based on Routine MDCT Imaging: A Preliminary Study on Patients with Vertebral Fractures

**DOI:** 10.1371/journal.pone.0116907

**Published:** 2015-02-27

**Authors:** Hans Liebl, Eduardo Grande Garcia, Fabian Holzner, Peter B. Noel, Rainer Burgkart, Ernst J. Rummeny, Thomas Baum, Jan S. Bauer

**Affiliations:** 1 Department of Radiology, Klinikum rechts der Isar, Technische Universitaet Muenchen, Muenchen, Germany; 2 Department of Orthopaedic Surgery, Klinikum rechts der Isar, Technische Universitaet Muenchen, Muenchen, Germany; 3 Section of Neuroradiology, Klinikum rechts der Isar, Technische Universitaet Muenchen, Muenchen, Germany; Faculté de médecine de Nantes, FRANCE

## Abstract

**Purpose:**

To experimentally validate a non-linear finite element analysis (FEA) modeling approach assessing in-vitro fracture risk at the proximal femur and to transfer the method to standard in-vivo multi-detector computed tomography (MDCT) data of the hip aiming to predict additional hip fracture risk in subjects with and without osteoporosis associated vertebral fractures using bone mineral density (BMD) measurements as gold standard.

**Methods:**

One fresh-frozen human femur specimen was mechanically tested and fractured simulating stance and clinically relevant fall loading configurations to the hip. After experimental in-vitro validation, the FEA simulation protocol was transferred to standard contrast-enhanced in-vivo MDCT images to calculate individual hip fracture risk each for 4 subjects with and without a history of osteoporotic vertebral fractures matched by age and gender. In addition, FEA based risk factor calculations were compared to manual femoral BMD measurements of all subjects.

**Results:**

In-vitro simulations showed good correlation with the experimentally measured strains both in stance (R^2^ = 0.963) and fall configuration (R^2^ = 0.976). The simulated maximum stress overestimated the experimental failure load (4743 N) by 14.7% (5440 N) while the simulated maximum strain overestimated by 4.7% (4968 N). The simulated failed elements coincided precisely with the experimentally determined fracture locations. BMD measurements in subjects with a history of osteoporotic vertebral fractures did not differ significantly from subjects without fragility fractures (femoral head: p = 0.989; femoral neck: p = 0.366), but showed higher FEA based risk factors for additional incident hip fractures (p = 0.028).

**Conclusion:**

FEA simulations were successfully validated by elastic and destructive in-vitro experiments. In the subsequent in-vivo analyses, MDCT based FEA based risk factor differences for additional hip fractures were not mirrored by according BMD measurements. Our data suggests, that MDCT derived FEA models may assess bone strength more accurately than BMD measurements alone, providing a valuable in-vivo fracture risk assessment tool.

## Introduction

Fragility fractures are a common complication of osteoporosis with severe associated risks. Osteoporotic fractures represent both an individual risk for the affected patient with significantly increased associated mortality as well as an economic burden for our ageing societies, due to the rising incidence of fragility fractures challenging already strained healthcare budgets [1]. Healthcare costs associated with osteoporotic fractures are estimated to amount to around 22 billion dollars every year in the United States [2]. Therefore the medical community strives to further investigate the etiopathology of ostoporosis as well as to develop new therapy options to counter the inherent challenges.

Accurate early diagnosis is key to successful treatment. Traditionally, dual energy X-ray absorptiometry (DXA) has been used to assess bone mineral density (BMD), thus identifying subjects at high risk for osteoporotic fractures. Areal bone mineral density as measured by DXA is an indicator for bone strength, which is known to show site-specific changes depending on age and gender [3–6]. Quantitative computed tomography (QCT) has been established as a sensitive technique to evaluate compartment specific, volumetric BMD [7]. However, all BMD measurements alone are limited by the fact that mineral density only partly accounts for bone strength, and other known influential factors for bone stability exist, such as bone geometry or the trabecular and cortical micro-architecture of the bone [8].

**Fig 1:**
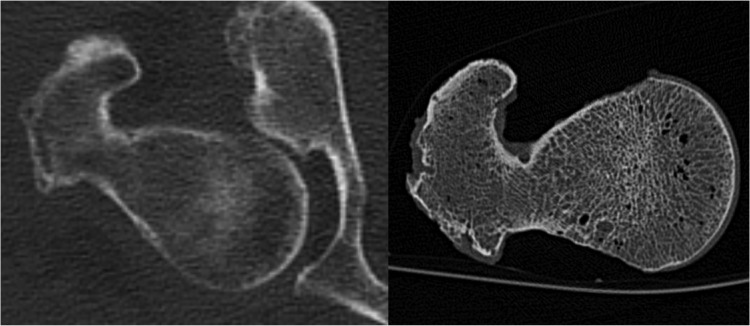
In vivo CT Image of a proximal femur with image noise and artifacts caused by surrounding soft tissue compared to an in vitro CT Image with better depiction of the trabecular and cortical structures. The spatial resolution, determined at ρ50 of the modulation-transfer-function, is 230μmx230μm *in vitro* and 250μmx250μm *in vivo*.

QCT is able to assess the three-dimensional (3D) bone geometry and bone density distribution providing input to finite element analysis (FEA) models in order to estimate bone strength in vitro [9–17]. Finite element analysis has been shown to predict stresses and strains throughout the bone related to the location and direction of impact forces, which are important determinants of fracture risk [17,18]. As a consequence, the in-vivo potential of numerical approaches based on QCT/FEA methods has been investigated to non-invasively assess fracture load, type and location [11,19]. However, for in-vivo application, major methodological challenges remain unsolved, since in vivo images are frequently impaired by the presence of soft tissue or motion artifacts, as demonstrated in Fig. 1 [20]. Furthermore the larger doses of radiation emitted by QCT as compared to DXA limit its broader applicability.

In clinical practice, multi-row detector computed tomography (MDCT) scans are routinely performed for a wide range of diagnostic indications. Using a specific calibration phantom, these MDCT scans may also be used to predict bone strength equivalent to QCT and subsequently assess fracture risk omitting the need for separate density scans [21]. This would potentially reduce radiation exposure, healthcare cost, time and effort particularly for patients with cancer, who routinely undergo MDCT examinations for staging, as they are often at increased risk for developing treatment related osteoporosis [22,23].

Therefore, the objective of the current study was to validate the numerical MDCT/FEA approach using an in-vitro experiment performed on a fresh frozen human femur, assessing experimentally measured failure load criteria based on imaging parameters. Furthermore we aimed to then apply the experimentally validated approach to assess the individual femoral fracture risk in 8 subjects with and without vertebral fragility fractures using femoral FEA modeling derived from in-vivo routine contrast-enhanced MDCT in correlation with femoral BMD measurements.

## Material and Methods

### In-vitro validation experiment

A human fresh-frozen femur from a 72 year-old male (size 1.56 m, weight 78 kg) was obtained from the Institute of pathology Klinikum Rechts der Isar. Written informed consent was obtained from the donor. The donor had dedicated his body for educational and research purposes prior to death, in compliance with local institutional and legislative requirements. The study was reviewed and approved by the local institutional review boards (Ethikkommission der Fakultaet fuer Medizin der Technischen Universitaet Muenchen, Germany; project number 5022/11). After an examination to exclude skeletal diseases, the bone was cleaned of soft tissue and degreased with ethanol. The femur was scanned using MDCT on a 256-row MDCT Scanner (iCT, Philips, Netherlands; exposure: 466 mA; voltage: 120 kV; interpolated voxel size: r150μm x 150μm; real spatial resolution determined at r50 of the modulation-transfer-function: 230μm x 230μm; slice thickness: 0.67 mm) with a dedicated osteoporosis calibration phantom (Mindways, San Francisco, CA, USA) to allow for conversion of the attenuation values (in Hounsfield Units, HU) to equivalent BMD (in milligrams calcium hydroxyl apatite per cubic centimeter).

**Fig 2:**
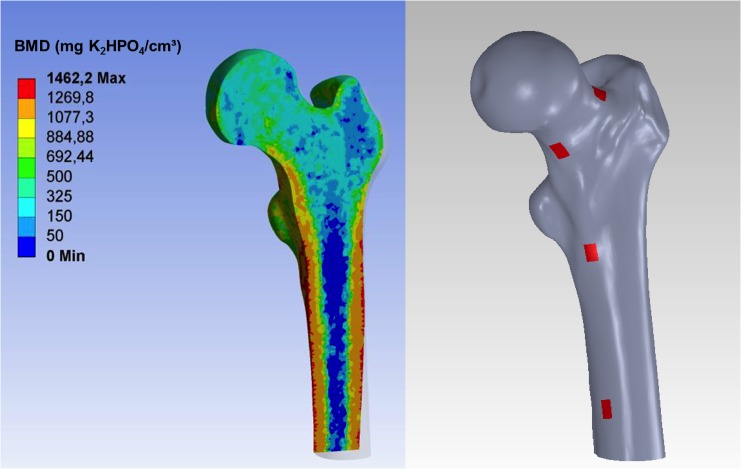
Representation of the bone mineral density distribution within the bone (left) and location of the string gauges at the femur specimen (right).

Thereafter the femur was distally trimmed, sealed and embedded up to 105 mm at the shaft as published by Besso et al. [24]. The shaft length between the lesser trochanter and the upper plane of the embedding resin measured twice the diameter of the femoral head. Furthermore epoxy resin (Rencast FC 52/53 Isocyanat and Polyol, Huntsman Group, Bad Saeckingen, Germany, pot life 3–4min, demoulding time 30–40min, surface temperature during embedding: 40°C) was used to improve the load transmission between the compression plate and the femoral head. To verify the model in the elastic range, 6 strain gauges were fixed on the surface of the bone as shown in Fig. 2. Five single strain gauges (Vishay CEA.06-062UR-350/P2, Vishay Precision Group, Malvern, USA) were used at the lower medial shaft, at the lesser and greater trochanter, and at the inferior and superior femoral neck. An additional strain gauge rosette (Vishay CEA.06-062UW-350/P2, Vishay Precision Group, Malvern, USA) was fixed to a site with minimal curvature at the femoral neck providing the strain field (i.e. principal strains) in that location. In order to validate the proposed FEA model, it was essential to precisely match the numerical boundary conditions with those of the experimental setup. For that purpose, the position of several points was acquired with a digitizer system (Microscribe 3DX, Immersion Corporation, USA) [25]: Positioning of the femoral bone using four CT markers at the femur, location and positioning of the strain gauges (SG, 3 vertexes), surface orientation of the embedding resin blocks (at the shaft and at the greater trochanter) as well as the position of the loading surface on the femoral head were assessed. The CT markers consisted of small metallic balls visible on the CT images, thus enabling calculation of the coordinate transformation between experiments and MDCT scans.

**Fig 3:**
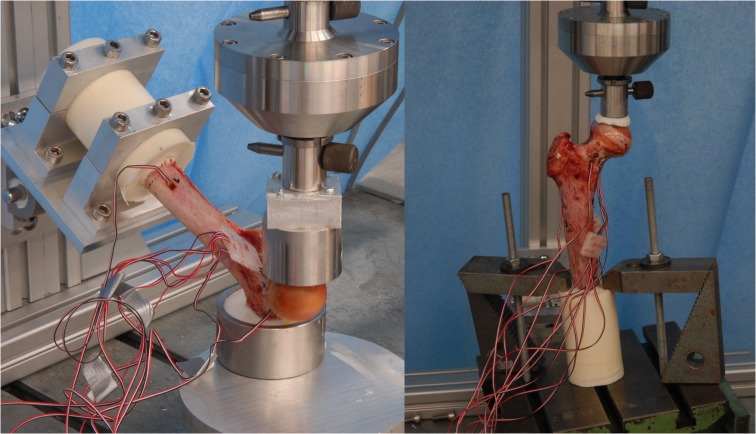
Experimental setup for the fall configuration (left) and the stance configuration.

**Fig 4:**
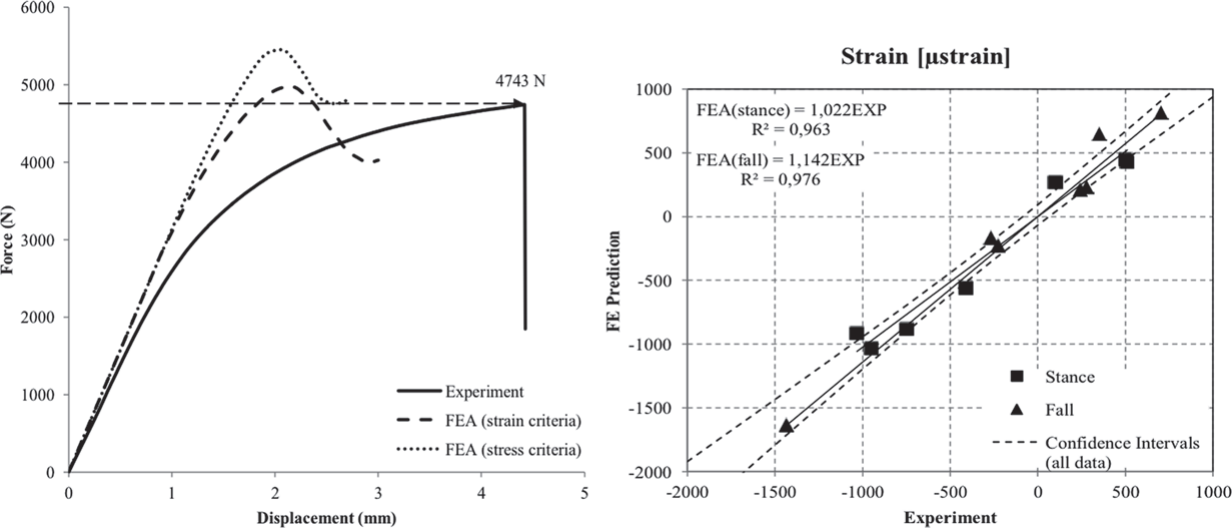
Left side: Load-displacement curves from the experimental data (failure load: 4743 N) as well as simulated forces extracted from the FEA models for both stress and strain criteria. Right side: Plotted FEA based predicted strains in both stance and fall configuration compared to experimentally measured values.

Subsequently, the specimen was tested under compression in a servo-electric testing machine (Wolpert TZZ 707/386, Wolpert GmbH; Instron, Massachusetts, USA) in two loading configurations: stance configuration and a sideways fall configuration as previously published [10]. For the stance configuration, the bone was placed with an inclination angle of 7° between loading direction and proximal shaft and loaded with incremented up to 1000 N by 5 mm/min [26]. For the fall configuration, the bone was placed with an inclination angle of 30° to the horizontal plane and internally rotated by 15° as suggested by Bessho, et al. and Grassi, et al. [13,27]. The velocity used for the load-to failure tests was set to 30 mm/min as published by Keyak et al. [28]. The greater trochanter was partially embedded and restrained. The distal end of the femur was attached to a fixture and could rotate about an axis perpendicular to the femur shaft (as shown in Fig. 3). In both configurations the load was applied to the femoral head transferred by a specifically made device, which allowed movement in the XY-plane, thus preventing the introduction of shear forces to the system. Finally, destructive fracture testing was performed as shown in Fig. 4 and the macroscopic aspect of the fracture was compared to the location of the failed elements yielded by the FEA model. Calculations of the simulated forces were then compared to the forces measured experimentally.

### In-vivo BMD assessment

The retrospective analyses of this study were conducted with approval of the Institutional Committee on Human Research (Ethikkommission der Fakultaet fuer Medizin der Technischen Universitaet Muenchen, Germany). Each patient had given written consent for scientific evaluation of their data including the analysis of medical images at the time of admission to our institution. Subject information was de-identified prior to analysis and stored anonymously. For this preliminary study a total of 8 subjects of western European descent who had recently received routine contrast-enhanced in-vivo MDCT scans at our institution to rule out tumor recurrence were selected from our digital picture archiving communication system (PACS, Sectra AB, Linkoeping, Sweden) as shown in Table 1. Four subjects with osteoporotic vertebral fragility fractures were identified by two radiologists in consensus and were matched by age and gender with 4 subjects with no history of fragility fracture. Further matching criteria were based on the MDCT scanning parameters (i.e. voxel size, exposure). Axial sections with a slice thickness of 0.67 mm as well as sagittal reformations with a slice thickness of 3 mm were used to assess vertebral fracture status according to the Genant classification by two radiologists in consensus [29,30]. Exclusion criteria were: orthopedic hardware or presence of metal artifacts; pathological bone changes other than osteoporosis in the femur such as fracture, osseous metastasis, circumscribed sclerosis (e.g. bone island), circumscribed lucencies (e.g. cystic lesions), hematological disorders or severe degeneration. All in-vivo contrast-enhanced MDCT examinations were performed with the same 256-row MDCT scanner (iCT, Philips, Netherlands) and scanning parameters as for the in-vitro experiment (interpolated voxel size 150x150x670μm^3^), except a slightly lower radiation dose (adapted tube load of averaged 200 mAs; real spatial resolution determined at r50 of the modulation-transfer-function: 250μmx250μm). The contrast-enhanced scans were performed after standardized administration of intravenous contrast medium (Imeron 400, Bracco, Konstanz, Germany) using a high-pressure injector (Fresenius Pilot C, Fresenius Kabi, Bad Homburg, Germany). The intravenous contrast medium injection was performed with a delay of 70 s, a flow rate of 3 mL/s, and a bodyweight-dependent dose (80 mL for body weight up to 80 kg, 90 mL for body weight up to 100 kg, and 100 mL for body weight over 100 kg). In addition, all patients were given 1,000 mL oral contrast medium (Barilux Scan, Sanochemia Diagnostics, Neuss, Germany). As previously published, contrast-enhanced MDCT image based BMD values may be corrected for the contrast effect with the following conversion equation: (areal) BMD = 0.99 × BMDMDCT—12 mg/cm^2^ [31]. However, as BMD was shown to deviate by less than 10% and is expected to change equally in all subjects, BMD was not converted for this study.

**Table 1:** Patient characteristics of the subjects from the fracture group and the control group and according force calculation values derived from the FEA simulations.

Subject	Gender	Vertebral Fracture	Age (years)	Height (in m)	Weight (in kg)	h cog (in cm)	F peak (in N)	Soft Tissue (in mm)	Soft Tissue attenuation (in N)	F atten (in N)	F sim (in N)
1	w	no	50	1.56	60	0.78	4073.96	36.2	2570.2	1503.76	3363.47
2	w	no	72	1.78	69	0.91	4666.74	35.0	2485.0	2181.74	3788.55
3	w	no	75	1.64	60	0.84	4177.12	29.4	2087.4	2089.71	3822.83
4	m	no	69	1.78	101	0.91	6364.23	36.2	2566.6	3797.58	5873.96
5	w	yes	47	1.68	64	0.86	4366.40	16.7	1185.7	3180.70	3261.43
6	w	yes	72	1.64	54	0.84	3962.76	23.8	1689.8	2272.96	3633.43
7	w	yes	76	1.58	80	0.81	4734.26	42.0	2982.0	1752.26	2343.27
8	m	yes	68	1.76	75	0.90	5453.34	26.7	1895.7	3557.64	4250.00

The acquired datasets were uploaded onto the institutional PACS (Sectra AB, Linkoeping, Sweden). As outlined previously, [32] circular regions of interest (ROIs) were placed at the femoral head in the section with the greatest diameter of the femoral head, covering as much of the trabecular compartment as possible while excluding any cortical structures. Additional rectangular ROIs were placed at the femoral neck in the section where the femoral neck was best visualized including the cortex imitating density measurements of the entire bone compartment similar to DXA assessments. Furthermore ROIs were placed in two reference regions within the designated bone density calibration phantom. Attenuation values (in HU) were then quantified from the ROIs using the density measurement tools of the PACS software. Attenuation values were then converted into BMD values representing the amount of calcium hydroxyl apatite in milligrams per cubic centimeter using the reference values from the calibration phantom, according to the guide lines of the manufacturer. A radiologist, who was blinded to the fracture status of the subjects, performed the placement of all ROIs as well as the calculation of BMD. The duration of ROI placement and BMD calculation was less than 2 min per subject.

### Fracture risk assessment using MDCT based finite element analysis (FEA)

Finite element models were built from the in-vitro femur data as well as from the in-vivo MDCT scans of the 8 subjects evaluated. Images were binarized and the bone contour smoothened using ImageJ (ImageJ, National Institutes of Health, USA). Semi automated segmentation with manual correction of the MDCT images enabled the generation of mathematical models (NURBS) of the bone surfaces using 3D tetrahedral 10-node meshes (SolidWorks, Dassault Systèmes SolidWorks Corp., USA). Different mesh refinements, element sizes and resulting strain energies were compared using ANSYS Workbench (ANSYS, Canonsburg, USA) under constant loading and boundary conditions yielding a suitable mesh protocol with maximum element edge lengths of 1.7 mm. Subsequently the MDCT images were converted into BMD maps assigning the according material properties to each corresponding node. The mapping procedure was performed using the voxel information (location and attenuation) saved as unique text files in combination with the values obtained from the reference regions provided by the calibration phantom. A subroutine was written for ANSYS to import the created text files and to assign the material properties node by node. Fig. 2 shows a visualization of the BMD distribution throughout the femur used for the in-vitro experiment. Subsequently, inhomogeneous isotropic elastic modulus E (in MPa) was calculated from the BMD (in mg/cm^3^) using the relationships presented in Table 2.

**Table 2:** Threshold values and property relationships adapted from the literature: Elastic property relationships for tensile and compressive testing, limit values for maximum stress and strain and threshold values used for bone mineral density.

Connotation Name	Property Relationship	Literature Reference
Elastic Modulus	E_trab_ (ρ_TRAB_) = 7607 * ρ_BMD_^1.853^ (BMD <500 mg cm^-3^)	Grande Garcia, 2012
Elastic Modulus	E_cort_ (ρ_ASH_) = 10200 * ρ_ASH_^2.01^	Keller, 1994
Ash Density	ρ_ASH_ = 1.220 ρ_QCT_ + 0.0526	Keyak et al., 2003
Maximum Principal Stress Limit Values	σ_yC_ (MPa) = 137 * ρ_ASH_^1.88^; ρ_ASH_ < 0.317 g cm^-3^	Keller, 1994; Keyak, et al., 2003
Maximum Principal Stress Limit Values	σ_yC_ (MPa) = 114 * ρ_ASH_^1.72^; ρ_ASH_ > 0.317 g cm^-3^	Keller, 1994; Keyak, et al., 2003
Tensile Yield	σ_yT_ = 0.8 * σ_yC_	Keller, 1994; Keyak, et al., 2003
Maximum Principal Strain Limit values	ϵ_yC_ = 0.0104 (compression)	Bayraktar, et al., 2004
Maximum Principal Strain Limit values	ϵ_yC_ = 0.0073 (tension)	Bayraktar, et al., 2004
Trabecular bone:	<500 mg K2HPO4 /cm³	Grande Garcia, 2012
Cortical bone:	>500 mg K2HPO4 /cm³	

The major difference between the modeling of the in-vitro and the in-vivo data was the definition of the boundary conditions. As previously described, the points of interest during the in-vitro experiments (i.e. MDCT markers, string gauges, constraints, loading conditions) were spatially registered using a digitizer and transferred to the FEA model using the corresponding CS transformation (coordinate system transformation). The loading direction for the in-vivo data aimed to reproduce the experimental setup using a simulated angle of 30° between the long axis of the femur shaft and a virtual horizontal plane with a rotation of 15° between the femoral neck axis and the horizontal plane. Displacements of selected nodes at the trochanter were set to zero in z-direction. A simulated displacement condition was then applied to the femoral head in z-direction. The distal surface of the femur shaft was constrained in displacement allowing only rotation about the x-axis.

In order to predict the fracture load of the bone to assess bone strength, an iterative bone damage model was required. Previous studies have determined the fracture load as the load at which at least one solid element suffers a minimum principal strain [24]. Such models are known to show string dependencies on mesh density and quality. A more intuitive approach is to determine the value of the failure load by measuring the peak value of the force-displacement curve. For our study, a yield model as published by Dragomir-Daescu et al. was created and slightly modified [15]. A simulated displacement boundary condition was applied to the defined loading surface on the femoral head in small increments. After each loading step, elements with a calculated risk factor greater than one were defined as failed elements by the assignment of a very small Young´s modulus (0.1 MPa). The stiffness model was then updated and solved repetitively in steps with increased displacement until the simulated load-displacement curve reached a maximum. To define the failing elements, two yield criteria (maximum stress, maximum strain) were used. For the evaluation of the in-vivo analysis results, the maximum strain criterion was selected. The tensile and compressive elastic limit values for maximum stress and strain were adopted from the literature as demonstrated in Table 2. To include only elements with high density for the analysis a BMD threshold value of 500 mg/cm^3^ was used as previously published to differentiate between cortical and trabecular bone [33,34].

### In-vitro/ in-vivo transfer

Previous studies [9–15] [11,19] have determined the validity of FEA modeling using femur specimens as outlined above. However, a remarkable aim of this work was to transfer the validated in-vitro method to the analysis of in-vivo data. In order to compare fracture load as calculated by FEA modeling between different subjects, an individual risk factor (Φ)for hip fracture was introduced. This risk factor was defined as the ratio of force applied to the hip during a sideways fall (F_peak_) to femoral strength (F_sim_) in a sideways fall configuration (Φ = Fpeak / Fsim). The effective peak force (F_peak_effec_) that would result at the hip during a sideways fall was estimated incorporating individual subject data such as the reported height and weight as well as force reduction due to cushioning by the surrounding soft tissue:

$$F_{peak\_effec}=F_{peak}–F_{att}=v*\surd (k_{stiff}*m/2)–(71*fat\_thickness)$$

The following equations were used: F_peak_ = v * √ (k_stiff_ * m/2) and v = √ (2 * g * h_cog_) where *v* is the velocity at time of impact, *g* is the gravitational constant (9.81 m/s²), *h*_*cog*_ is the height of an individual´s center of gravity simulating a fall from standing height, m is the effective mass (kg), and *k*_*stiff*_ is the sex-specific stiffness constant (N/m). The sex-specific stiffness constant (k_stiff_ women: 35442 Nm; k_stiff_ men: 45031 Nm) published by Bouxsein et al. and Nielson et al. [35,36] has been specifically calculated for a sideways fall scenario and incorporates a variety of gender specific and individual patient characteristics such as age, height, weight, etc. [36,37]. Furthermore the attenuated force (F_att_) was computed accounting for the reduction of the peak force (F_peak_) by surrounding peritrochanteric soft tissue including cushioning muscle and fat. The reduction of the peak force applied to the hip was calculated in our experimental model to be 71 N per 1 mm of covering tissue thickness. The thickness measurements of the trochanteric soft tissue cover were derived from in-vivo CT images assessing the shortest distance between the greater trochanter and the outer patient contour parallel to the patient axis, as previously described in the literature [37–39].

### Statistical Analysis

Due to the limited subject number included in this proof of principle study, statistical analysis was limited. Femoral BMD as well as risk factor values of subjects with and without vertebral fragility fractures were compared using t-tests. A Kolmogorov-Smirnov test and a Shapiro-Wilk test designed for a small sample size showed no significant difference from a normal distribution (p>0.05) for all parameters. In addition, a Wilcoxon signed rank test was used due to the small sample size. As no significant differences to the results of the t-test were found, only these p-values were reported in the paper. A significance level of p<0.05 was used for all analyses. Follow-up studies are needed to validate the initial findings to allow for extrapolation of the hypothesis with application to larger cohorts.

## Results

### In-vitro analysis

The in-vitro experiment showed that the FEA models provided realistic predictions of the elastic regimen of the femur for both loading configurations. The predicted strains correlated with the measured strains both in the stance configuration (R² = 0.963, slope 1.022) and in the fall configuration (R² = 0.976, slope 1.142). In both simulations the model predicted slightly higher bone strength than measured by experimental testing. The maximum points of the force-displacement curves estimated predicted failure load values. Both failure criteria generated slightly overestimated results as compared to the experimental measurements: The max stress criterion simulation overestimated the experimental failure load (4743 N) by 14.7% (5440 N) while the max strain criterion simulation performed more accurate overestimating by 4.7% (4968 N). Fig. 4 illustrates force displacement curves demonstrating FEA based simulated force calculations and experimentally measured values extracted from the mechanical testing machine along with plotted force values in both loading configurations.

**Fig 5:**
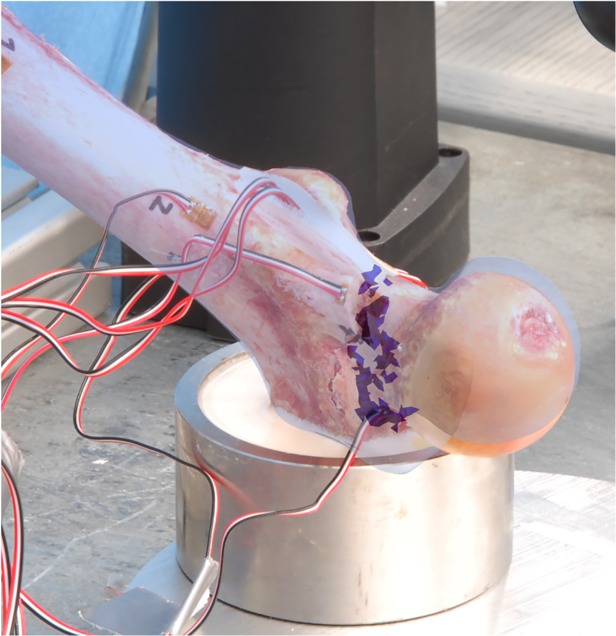
Image overlay of the macroscopic aspect of the fractured bone after destructive testing and the failed elements derived from the FE model (right). Failed elements derived from the FEA model are marked in purple, the change in shape (dislocation after destructive testing) is illustrated as blue shadow.

In addition, the failed elements as simulated by the FEA model were localized visually to evaluate the simulated fracture location. Subsequently the experimentally determined fracture location was compared to the predicted fracture site. As illustrated in Fig. 5, the visual evaluation of the simulated and the experimentally determined fracture locations at the femoral neck coincided precisely.

### In vivo analysis

Data for the in-vivo FEA force simulations as well as characteristics of the imaging datasets from the 8 subjects analyzed are presented in Table 1. The method validated in-vitro was transferred to in-vivo imaging data of 8 subjects as described above, calculating an individual risk factor (RF Φ) for fracture risk at the hip based on MDCT derived FEA analysis. FEA derived risk factor values along with according BMD measurements of the femoral head and neck are presented in Table 3, showing the 4 subjects with a history of osteoporotic vertebral fracture to present with a higher risk factor for incident hip fracture as compared to the matched control subjects without history of vertebral fragility fracture (p = 0.028). According BMD measurements from the femoral head and neck of these 8 subjects were compared to the calculated risk factor values as outline in Table 3. Unlike the risk factor assessment from the FEA model, BMD measurements of the hip did not show significant differences (femoral head: p = 0.989; femoral neck: p = 0.366) between the subjects depending on vertebral fracture status.

**Table 3:** Risk factor values calculated for each subject based on FEA simulations presented along with according in-vivo BMD (in mg/cm^3^) measurements of the femoral head and neck.

Subject	Gender	Vertebral Fracture	Risk Factor	BMD (Femoral Head)	BMD (Femoral Neck)
1	female	no	0.45	278.58	183.74
2	female	no	0.58	192.54	139.39
3	female	no	0.55	149.81	162.00
4	male	no	0.65	202.11	171.51
5	female	yes	0.98	179.88	147.23
6	female	yes	0.63	213.94	194.61
7	female	yes	0.75	244.23	126.64
8	male	yes	0.84	186.64	104.77
Average (Subjects without Fractures)			0.55	205.76	164.16
Average (Subjects with Fractures)			0.80	206.17	143.31

## Discussion

In this study finite element analysis derived from standard MDCT of a human femur were validated with in-vitro experiments to assess bone strength simulating a physiological stance configuration as well as a sideways fall to the hip. The experimentally validated method was subsequently transferred to a set of in-vivo MDCT data to calculate an individualized FEA based risk factor for hip fracture, and to compare the calculated risk factor to according BMD measurements of the femoral head and neck. The FEA derived risk factor for hip fracture demonstrated differences between subjects with a history of vertebral fragility fracture versus healthy, age matched controls, that were not mirrored in the according BMD assessments.

Biomechanical testing represents the gold standard to validate the simulated mechanical properties of different loading configurations such as loading and falling. Bessho et al. performed a study investigating different loading and boundary conditions at the proximal femur and showed that simulated FEA fracture type and experimentally determined fracture location coincided [11]. Koivumaeki et. al. performed thorough in-vitro testing of femur cadavers to validate FEA models derived from CT imaging, showing that FEA reliably predicts fracture load in a sideways fall configuration.[14] However, few studies have used in-vivo data, or transferred in-vitro to in-vivo data. Our experiment shows coincidence of the simulated and the experimentally determined fracture load, as well as congruence of the simulated and experimentally observed fracture location indicating a successful in-vivo application of the method tested in-vitro.

Various studies have demonstrated, that FEA derived biomechanical simulation may prove more reliable in assessing bone stability than conventional density measurements. In an extensive review over multiple studies, Zysset et al. recently analyzed the role of FEA in the past decades at the main fracture sites for osteoporotic fragility fractures and concluded, that FEA provides the most reliable surrogates of bone strength compared to any of the conventionally used densitometric standards [18]. Dall’Ara recently published an in-vitro study showing that QCT-based nonlinear FE models do not only predict mechanical properties at the femur better than densitometry, but also showed FEA to provide useful additional qualitative information about failure location [10]. In addition, Graeff et al. demonstrated hr-QCT derived FEA variables to be more accurate in distinguishing patients of differing vertebral fracture status as compared to conventional DXA measurements [8]. In accordance with these findings, our study observed a difference in the FEA based calculated risk factor for hip fracture, when analyzing subjects with and without vertebral fractures. The difference observed was not mirrored by the according BMD measurements encouraging further validation of such an FEA based risk factor. FEA may not only be useful for fracture prediction: In another study by Graeff et al., treatment related changes in vertebral bone could be monitored more accurately using FE analysis when longitudinally analyzing osteoporotic women receiving teriparatide treatment [40]. In addition, many of the advanced approaches to characterize bone structure and strength beyond BMD are limited to the periphery of the skeleton [41,42]. However, substantial heterogeneity of bone strength among the different clinically relevant skeletal sites has been reported, as published by Eckstein et al. [43]. Therefore direct bone strength assessments at the relevant sites such as in-vivo FEA of the proximal femur may perform best in predicting fracture risk.

To this day, femoral and vertebral strength measurements cannot be reliably obtained from MRI imaging, despite considerable efforts [44–46]. Unfortunately, radiation exposure for high resolution evaluation of the trabecular structure of the axial skeleton is considerable, and has been reported to reach up to 3 mSv for HR-QCT of the spine, limiting broad applicability [40]. However, as demonstrated in the current study, FE analysis can also be derived from clinical routine MDCT. This potentially redundantizes additional bone density assessments particularly for cancer patients, who are at increased risk for osteoporotic fractures due to chemotherapy, hormone therapy, e.g. women with breast cancer, men with prostate cancer, or adults treated for hematologic disorders [47,48]. Care has to be taken when using routine MDCT imaging, to take into account the influence of intravenous contrast medium on FEA as well as BMD. Baum et al. have shown BMD to increase considerably when analyzing contrast-enhanced MDCT scans [49]. Nevertheless, there is growing interest in applying FEA analysis for clinical application, as with improving hardware systems (e.g. flat panel CT systems) [50] and more refined post processing algorithms such as iterative reconstruction, radiation exposure may be significantly lowered [51,52]. Therefore various recent studies focus on investigating bone strength using finite element analysis [53]. Some recent studies even suggest the future use of reconstructed conventional radiographs for finite element analysis [54].

Nevertheless, there are some limitations resulting from the study design regarding the quality of the CT data, the FE modeling and the calculation of the applied forces. Because the in-vivo data was derived from routine MDCT, the exposure and resulting resolution was lower compared to the in-vitro specimen CT images. Additional filtering (Median filter; ImageJ) was used to reduce image noise and to enable meaningful segmentation and the influence of the surrounding soft tissue on image quality and the material property assignments was accounted for to the extent possible. Another limitation results from the contingency of the force in a fall configuration on the stiffness properties (k_stiff_) of the femur, which must not be equated with the simulated stiffness of the proximal femur. After extensive review of the literature, a direct reference to the value k_stiff_ could not be found and consequently sex-specific stiffness constant values were adopted from the literature as published by Bouxsein et al. and Nielson et al. [35,36]. Also, significant computational resources are needed for FEA analysis. Koivumaeki et al. investigated the use of simplified finite element models requiring less computational power, requiring less time and computer hardware [55]. Furthermore the small cohort size of the in-vivo subject group and the availability of one femur specimen only for the ex-vivo experiment limit meaningful statistical analysis and interpretation. Therefore the plausibility of the results of this proof-of-principle study should be further evaluated in a larger frame also comparing CT based FEA to conventional DXA.

## Conclusion

This study was designed as an experimental proof of principle study to evaluate the feasibility to assess bone strength using non-linear homogenized FEA derived from standard clinical routine MDCT imaging. FEA simulations were successfully validated by destructive in-vitro testing of a human femoral cadaver specimen. Subsequently the FEA method was applied to in-vivo MDCT images demonstrating elevated risk factor values for additional hip fracture in subjects presenting with vertebral fractures. The data of this study suggests, that FEA based risk factor calculations may predict fracture risk in subjects with and without vertebral osteoporotic fractures more accurately than BMD measurements alone. MDCT derived FEA models therefore may provide a valuable in-vivo fracture risk assessment tool with the potential for clinical applicability. Further studies are needed to investigate whether MDCT based FEA models can accurately predict fracture risk in osteoporotic subjects.

## References

[1] Bliuc D, Nguyen ND, Milch VE, Nguyen TV, Eisman JA, et al (2009) Mortality risk associated with low-trauma osteoporotic fracture and subsequent fracture in men and women. JAMA 301: 513–521. 10.1001/jama.2009.50 19190316

[2] Blume SW, Curtis JR (2011) Medical costs of osteoporosis in the elderly Medicare population. Osteoporos Int 22: 1835–1844. 10.1007/s00198-010-1419-7 21165602 PMC3767374

[3] McCreadie BR, Goldstein SA (2000) Biomechanics of fracture: is bone mineral density sufficient to assess risk? J Bone Miner Res 15: 2305–2308. 11127195 10.1359/jbmr.2000.15.12.2305

[4] Eckstein F, Matsuura M, Kuhn V, Priemel M, Muller R, et al (2007) Sex differences of human trabecular bone microstructure in aging are site-dependent. J Bone Miner Res 22: 817–824. 17352643 10.1359/jbmr.070301

[5] Greenspan SL, Maitland-Ramsey L, Myers E (1996) Classification of osteoporosis in the elderly is dependent on site-specific analysis. Calcif Tissue Int 58: 409–414. 8661481 10.1007/BF02509439

[6] Bjarnason K, Hassager C, Svendsen OL, Stang H, Christiansen C (1996) Anteroposterior and lateral spinal DXA for the assessment of vertebral body strength: comparison with hip and forearm measurement. Osteoporos Int 6: 37–42. 8845598 10.1007/BF01626536

[7] Bousson V, Le Bras A, Roqueplan F, Kang Y, Mitton D, et al (2006) Volumetric quantitative computed tomography of the proximal femur: relationships linking geometric and densitometric variables to bone strength. Role for compact bone. Osteoporos Int 17: 855–864. 16547689 10.1007/s00198-006-0074-5

[8] Graeff C, Marin F, Petto H, Kayser O, Reisinger A, et al (2013) High resolution quantitative computed tomography-based assessment of trabecular microstructure and strength estimates by finite-element analysis of the spine, but not DXA, reflects vertebral fracture status in men with glucocorticoid-induced osteoporosis. Bone 52: 568–577. 10.1016/j.bone.2012.10.036 23149277

[9] Dall'Ara E, Pahr D, Varga P, Kainberger F, Zysset P (2012) QCT-based finite element models predict human vertebral strength in vitro significantly better than simulated DEXA. Osteoporos Int 23: 563–572. 10.1007/s00198-011-1568-3 21344244

[10] Dall'Ara E, Luisier B, Schmidt R, Kainberger F, Zysset P, et al (2013) A nonlinear QCT-based finite element model validation study for the human femur tested in two configurations in vitro. Bone 52: 27–38. 10.1016/j.bone.2012.09.006 22985891

[11] Bessho M, Ohnishi I, Matsumoto T, Ohashi S, Matsuyama J, et al (2009) Prediction of proximal femur strength using a CT-based nonlinear finite element method: differences in predicted fracture load and site with changing load and boundary conditions. Bone 45: 226–231. 10.1016/j.bone.2009.04.241 19398043

[12] Yosibash Z, Tal D, Trabelsi N (2010) Predicting the yield of the proximal femur using high-order finite-element analysis with inhomogeneous orthotropic material properties. Philos Trans A Math Phys Eng Sci 368: 2707–2723. 10.1098/rsta.2010.0074 20439270

[13] Grassi L, Schileo E, Taddei F, Zani L, Juszczyk M, et al (2012) Accuracy of finite element predictions in sideways load configurations for the proximal human femur. J Biomech 45: 394–399. 10.1016/j.jbiomech.2011.10.019 22079387

[14] Koivumaki JE, Thevenot J, Pulkkinen P, Kuhn V, Link TM, et al (2012) Ct-based finite element models can be used to estimate experimentally measured failure loads in the proximal femur. Bone 50: 824–829. 10.1016/j.bone.2012.01.012 22306697

[15] Dragomir-Daescu D, Op Den Buijs J, McEligot S, Dai Y, Entwistle RC, et al (2011) Robust QCT/FEA models of proximal femur stiffness and fracture load during a sideways fall on the hip. Ann Biomed Eng 39: 742–755. 10.1007/s10439-010-0196-y 21052839 PMC3870095

[16] Crawford RP, Rosenberg WS, Keaveny TM (2003) Quantitative computed tomography-based finite element models of the human lumbar vertebral body: effect of element size on stiffness, damage, and fracture strength predictions. J Biomech Eng 125: 434–438. 12968567 10.1115/1.1589772

[17] Crawford RP, Cann CE, Keaveny TM (2003) Finite element models predict in vitro vertebral body compressive strength better than quantitative computed tomography. Bone 33: 744–750. 14555280 10.1016/s8756-3282(03)00210-2

[18] Zysset PK, Dall'ara E, Varga P, Pahr DH (2013) Finite element analysis for prediction of bone strength. Bonekey Rep 2: 386 10.1038/bonekey.2013.120 24422106 PMC3765052

[19] Cody DD, Hou FJ, Divine GW, Fyhrie DP (2000) Short term in vivo precision of proximal femoral finite element modeling. Ann Biomed Eng 28: 408–414. 10870897 10.1114/1.278

[20] Cheung AM, Adachi JD, Hanley DA, Kendler DL, Davison KS, et al (2013) High-resolution peripheral quantitative computed tomography for the assessment of bone strength and structure: a review by the Canadian Bone Strength Working Group. Curr Osteoporos Rep 11: 136–146. 10.1007/s11914-013-0140-9 23525967 PMC3641288

[21] Baum T, Grabeldinger M, Rath C, Grande Garcia E, Burgkart R, et al (2014) Trabecular bone structure analysis of the spine using clinical MDCT: can it predict vertebral bone strength? J Bone Miner Metab 32: 56–64. 10.1007/s00774-013-0465-6 23604586

[22] Gralow JR, Biermann JS, Farooki A, Fornier MN, Gagel RF, et al (2009) NCCN Task Force Report: Bone Health in Cancer Care. J Natl Compr Canc Netw 7 Suppl 3: S1–32; quiz S33–35. 19555589 10.6004/jnccn.2009.0076 PMC3047404

[23] Guise TA (2006) Bone loss and fracture risk associated with cancer therapy. Oncologist 11: 1121–1131. 17110632 10.1634/theoncologist.11-10-1121

[24] Bessho M, Ohnishi I, Matsuyama J, Matsumoto T, Imai K, et al (2007) Prediction of strength and strain of the proximal femur by a CT-based finite element method. J Biomech 40: 1745–1753. 17034798 10.1016/j.jbiomech.2006.08.003

[25] Taddei F, Schileo E, Helgason B, Cristofolini L, Viceconti M (2007) The material mapping strategy influences the accuracy of CT-based finite element models of bones: an evaluation against experimental measurements. Med Eng Phys 29: 973–979. 17169598 10.1016/j.medengphy.2006.10.014

[26] Yosibash Z, Trabelsi N, Milgrom C (2007) Reliable simulations of the human proximal femur by high-order finite element analysis validated by experimental observations. J Biomech 40: 3688–3699. 17706228 10.1016/j.jbiomech.2007.06.017

[27] Bessho M, Ohnishi I, Okazaki H, Sato W, Kominami H, et al (2004) Prediction of the strength and fracture location of the femoral neck by CT-based finite-element method: a preliminary study on patients with hip fracture. J Orthop Sci 9: 545–550. 16228668 10.1007/s00776-004-0824-1

[28] Keyak JH, Rossi SA, Jones KA, Les CM, Skinner HB (2001) Prediction of fracture location in the proximal femur using finite element models. Med Eng Phys 23: 657–664. 11755810 10.1016/s1350-4533(01)00094-7

[29] Genant HK, Wu CY, van Kuijk C, Nevitt MC (1993) Vertebral fracture assessment using a semiquantitative technique. J Bone Miner Res 8: 1137–1148. 8237484 10.1002/jbmr.5650080915

[30] Bauer JS, Muller D, Ambekar A, Dobritz M, Matsuura M, et al (2006) Detection of osteoporotic vertebral fractures using multidetector CT. Osteoporos Int 17: 608–615. 16437195 10.1007/s00198-005-0023-8

[31] Bauer JS, Henning TD, Mueller D, Lu Y, Majumdar S, et al (2007) Volumetric quantitative CT of the spine and hip derived from contrast-enhanced MDCT: conversion factors. AJR Am J Roentgenol 188: 1294–1301. 17449773 10.2214/AJR.06.1006

[32] Gruber M, Bauer JS, Dobritz M, Beer AJ, Wolf P, et al (2013) Bone mineral density measurements of the proximal femur from routine contrast-enhanced MDCT data sets correlate with dual-energy X-ray absorptiometry. Eur Radiol 23: 505–512. 10.1007/s00330-012-2629-5 22932742

[33] Keller TS (1994) Predicting the compressive mechanical behavior of bone. J Biomech 27: 1159–1168. 7929465 10.1016/0021-9290(94)90056-6

[34] Bayraktar HH, Morgan EF, Niebur GL, Morris GE, Wong EK, et al (2004) Comparison of the elastic and yield properties of human femoral trabecular and cortical bone tissue. J Biomech 37: 27–35. 14672565 10.1016/s0021-9290(03)00257-4

[35] Nielson CM, Bouxsein ML, Freitas SS, Ensrud KE, Orwoll ES, et al (2009) Trochanteric soft tissue thickness and hip fracture in older men. J Clin Endocrinol Metab 94: 491–496. 10.1210/jc.2008-1640 19017753 PMC2646514

[36] Bouxsein ML, Szulc P, Munoz F, Thrall E, Sornay-Rendu E, et al (2007) Contribution of trochanteric soft tissues to fall force estimates, the factor of risk, and prediction of hip fracture risk. J Bone Miner Res 22: 825–831. 17352651 10.1359/jbmr.070309

[37] Roberts BJ, Thrall E, Muller JA, Bouxsein ML (2010) Comparison of hip fracture risk prediction by femoral aBMD to experimentally measured factor of risk. Bone 46: 742–746. 10.1016/j.bone.2009.10.020 19854307

[38] van den Kroonenberg AJ, Hayes WC, McMahon TA (1996) Hip impact velocities and body configurations for voluntary falls from standing height. J Biomech 29: 807–811. 9147979 10.1016/0021-9290(95)00134-4

[39] Imai K, Ohnishi I, Bessho M, Nakamura K (2006) Nonlinear finite element model predicts vertebral bone strength and fracture site. Spine (Phila Pa 1976) 31: 1789–1794. 16845352 10.1097/01.brs.0000225993.57349.df

[40] Graeff C, Chevalier Y, Charlebois M, Varga P, Pahr D, et al (2009) Improvements in vertebral body strength under teriparatide treatment assessed in vivo by finite element analysis: results from the EUROFORS study. J Bone Miner Res 24: 1672–1680. 10.1359/jbmr.090416 19419306

[41] Baum T, Karampinos DC, Liebl H, Rummeny EJ, Waldt S, et al (2013) High-resolution bone imaging for osteoporosis diagnostics and therapy monitoring using clinical MDCT and MRI. Curr Med Chem 20: 4844–4852. 24083607 10.2174/09298673113206660279

[42] Kazakia GJ, Hyun B, Burghardt AJ, Krug R, Newitt DC, et al (2008) In vivo determination of bone structure in postmenopausal women: a comparison of HR-pQCT and high-field MR imaging. J Bone Miner Res 23: 463–474. 18052756 10.1359/jbmr.071116

[43] Eckstein F, Lochmuller EM, Lill CA, Kuhn V, Schneider E, et al (2002) Bone strength at clinically relevant sites displays substantial heterogeneity and is best predicted from site-specific bone densitometry. J Bone Miner Res 17: 162–171. 11771664 10.1359/jbmr.2002.17.1.162

[44] Zhang N, Magland JF, Rajapakse CS, Bhagat YA, Wehrli FW (2013) Potential of in vivo MRI-based nonlinear finite-element analysis for the assessment of trabecular bone post-yield properties. Med Phys 40: 052303 10.1118/1.4802085 23635290 PMC3651211

[45] Newitt DC, Majumdar S, van Rietbergen B, von Ingersleben G, Harris ST, et al (2002) In vivo assessment of architecture and micro-finite element analysis derived indices of mechanical properties of trabecular bone in the radius. Osteoporos Int 13: 6–17. 11878456 10.1007/s198-002-8332-0

[46] Chang G, Rajapakse CS, Babb JS, Honig SP, Recht MP, et al (2012) In vivo estimation of bone stiffness at the distal femur and proximal tibia using ultra-high-field 7-Tesla magnetic resonance imaging and micro-finite element analysis. J Bone Miner Metab 30: 243–251. 10.1007/s00774-011-0333-1 22124539 PMC3723134

[47] Yamamoto DS, Viale PH (2009) Update on identifying and managing osteoporosis in women with breast cancer. Clin J Oncol Nurs 13: E18–29. 10.1188/09.CJON.E18-E29 19793700

[48] Smith MR (2006) Treatment-related osteoporosis in men with prostate cancer. Clin Cancer Res 12: 6315s–6319s. 17062721 10.1158/1078-0432.CCR-06-0846 PMC3047394

[49] Baum T, Muller D, Dobritz M, Rummeny EJ, Link TM, et al (2011) BMD measurements of the spine derived from sagittal reformations of contrast-enhanced MDCT without dedicated software. Eur J Radiol 80: e140–145. 10.1016/j.ejrad.2010.08.034 20851544

[50] Mulder L, van Rietbergen B, Noordhoek NJ, Ito K (2012) Determination of vertebral and femoral trabecular morphology and stiffness using a flat-panel C-arm-based CT approach. Bone 50: 200–208. 10.1016/j.bone.2011.10.020 22057082

[51] Noel PB, Fingerle AA, Renger B, Munzel D, Rummeny EJ, et al (2011) Initial performance characterization of a clinical noise-suppressing reconstruction algorithm for MDCT. AJR Am J Roentgenol 197: 1404–1409. 10.2214/AJR.11.6907 22109296

[52] Noel PB, Renger B, Fiebich M, Munzel D, Fingerle AA, et al (2013) Does iterative reconstruction lower CT radiation dose: evaluation of 15,000 examinations. PLoS One 8: e81141 10.1371/journal.pone.0081141 24303035 PMC3841128

[53] Kopperdahl DL, Aspelund T, Hoffmann PF, Sigurdsson S, Siggeirsdottir K, et al (2014) Assessment of incident spine and hip fractures in women and men using finite element analysis of CT scans. J Bone Miner Res 29: 570–580. 10.1002/jbmr.2069 23956027 PMC3925753

[54] Thevenot J, Koivumaki J, Kuhn V, Eckstein F, Jamsa T (2014) A novel methodology for generating 3D finite element models of the hip from 2D radiographs. J Biomech 47: 438–444. 10.1016/j.jbiomech.2013.11.004 24290135

[55] Koivumaki JE, Thevenot J, Pulkkinen P, Kuhn V, Link TM, et al (2012) Cortical bone finite element models in the estimation of experimentally measured failure loads in the proximal femur. Bone 51: 737–740. 10.1016/j.bone.2012.06.026 22796418

